# Seroprevalence of *Toxoplasma gondii* in Women Who Have Aborted in Comparison with the Women with Normal Delivery in Ahvaz, Southwest of Iran

**DOI:** 10.1155/2015/764369

**Published:** 2015-01-29

**Authors:** J. Saki, N. Mohammadpour, F. Moramezi, S. Khademvatan

**Affiliations:** ^1^Department of Medical Parasitology and Health Research Institute, Infectious and Tropical Diseases Research Center, Ahvaz Jundishapur University of Medical Sciences, Ahvaz, Iran; ^2^Department of Medical Parasitology, Ahvaz Jundishapur University of Medical Sciences, Ahvaz, Iran; ^3^Department of Gynecology, Imam Khomeini Hospital, Ahvaz Jundishapur University of Medical Sciences, Ahvaz, Iran; ^4^Department of Medical Parasitology and Mycology & Social Determinants of Health Research Center, Urmia University of Medical Sciences, Urmia, Iran; ^5^Cellular & Molecular Research Center, Jundishapur University of Medical Science, Ahvaz, Iran

## Abstract

*Toxoplasma gondii* is an obligate intracellular protozoan parasite causing toxoplasmosis in animals and humans. Primary maternal infection with toxoplasmosis during pregnancy is frequently associated with transplacental transmission to the fetus. However it is not certain whether *Toxoplasma* infection can cause recurrent abortion. The aim of this study was to determine the relationship between *Toxoplasma* infection and abortion via detection of anti-*Toxoplasma gondii* antibodies in sera of women with obstetrical problems and compare the results with control group consisting of women with history of normal delivery. Sera from 130 women with abortion and sera of 130 women with normal delivery were tested for IgG and IgM anti-*Toxoplasma gondii* antibodies by ELISA method. The present study revealed 24.6% of the samples with abortion and 21.5% of the samples with normal delivery were positive for IgG antibodies. However, statistical analysis indicated no significant differences (*P* > 0.05). In addition, IgM antibody was detected in one woman who had aborted but not in women with normal childbirth. This study showed no significant difference between the case and control groups in IgG anti-*Toxoplasma* antibody but detected one sample with IgM antibodies in woman with abortion during the first trimester of pregnancy. In order to determine the relationship between *Toxoplasma* infection and abortion, anti-*Toxoplasma* IgG avidity and PCR to discriminate between recent and prior infections are recommended.

## 1. Introduction

*Toxoplasma gondii* is an obligate intracellular protozoan parasite responsible for animal and human toxoplasmosis and one of the most common chronic diseases affecting one-third of the world's human population [1]. The seroepidemiological evaluations indicate that toxoplasmosis is one of the most prevalent human diseases in many countries [2]. Transmission of* T. gondii* is usually by ingestion of cysts infected and undercooked or raw meat or by accidental ingestion of oocysts that may contaminate soil, water, and food. Meat is one of the most important sources of the infection in individuals [3].

Toxoplasmosis is also one of the infections that can be transmitted through placenta during pregnancy [2]. Although toxoplasmosis is largely asymptomatic in the majority of women, primary infection during pregnancy can result in disease transmission through the placenta and lead to hazardous consequences such as abortion, stillbirth, different degrees of mental or physical retardation, hydrocephalus, and blindness [2, 4, 5]. The seroprevalence of* T. gondii* antibodies in pregnant women can vary from 6.1 to 75.2% based on the geographical region [2]. Congenital toxoplasmosis can occur as a primary infection acquired during pregnancy [6], but not from the reactivation of a latent infection in immune competent pregnant women [7]. Several studies have suggested the role of* T. gondii* in the causation of abortions. Several serological assays have detected the immunoglobulin (IgG and IgM) antibodies against* T. gondii* in the serum. Among the assays, ELISA shows high sensitivity and specificity. In Ahvaz city southwest Iran, the incidence of IgG and IgM anti-*Toxoplasma* antibodies in women with high risk pregnancies and habitual abortion has not been documented. Therefore the presence of anti-*T. gondii* antibodies in cases of normal delivery and abortions, referred to the Teaching Hospital of Ahvaz Jundishapur University of Medical Sciences, was investigated, with the aim to determine the relationship between* Toxoplasma* infection and abortion.

## 2. Materials and Methods

The women admitted to the Gynecology Clinic of Ahvaz Imam Khomeini Teaching Hospital, affiliated to Jundishapur University of Medical Sciences, from April 2012 to March 2013 were included in this case control study. In this study the blood samples were prepared from two groups (study group and control group). The study group were women who had been referred to hospital because of abortion and for treatment and the control group were women who had normal delivery and were referred to hospital for a checkup.

The blood samples were collected from case group (patients) including 130 women with abortion and from the control group including 130 women with normal delivery and serum separated. ELISA (Trinity, Biotech, USA) was used for detection of anti-*Toxoplasma* IgG and IgM antibodies in the case and control groups. The results were analyzed using the SPSS software version 16 and *t*-test and chi square statistical tests. Value less than 0.05 was considered as statistically significant.

## 3. Results and Discussion

Out of 130 women with abortion, 32 (24.6%) and, out of 130 women with normal delivery, 28 (21.5%) were positive for IgG antibody against* T. gondii*. However, statistical analysis indicated no significant differences (*P* > 0.05). In addition, IgM antibody was detected in one woman who had aborted but not in women with normal childbirth. Comparison of average antibody titer in the case and control groups showed no statistical significant differences (*P* > 0.05). In regard to the trimesters of pregnancy, for the case group during the first trimester of pregnancy there was 8/30 (26.66%) IgG seropositive, and 1/30 (3.33%) was IgM positive; in the second trimester 6/40 (15%) were IgG positive and in the third trimester of pregnancy 17/60 (28.3%) were IgG positive. No samples of second and third trimesters of the case group had IgM positive results. For the control group during the first trimester of pregnancy there was 7/33 (21.21%) IgG seropositive; in the second trimester 5/37 (13.51%) were IgG positive and in the third trimester of pregnancy 16/62 (25.8%) were IgG positive. No samples of the control group had IgM positive results.

Out of 32 positive cases, 19 and 13 patients were urban and rural, respectively.

Early diagnosis of acute toxoplasmosis during pregnancy is needed for assessment of vertical transmission risk of infections and prevention of related severe complications.

Seroprevalence study showed* Toxoplasma* infection in pregnant women is between 7 and 51.3% throughout the world and the results of anti-*Toxoplasma* antibodies in women with abnormal pregnancy varied from 17.5 to 52.3% [8].

In women with acute toxoplasmosis,* Toxoplasma* transmission rate through placenta in the first, second, and third trimesters is 25, 54, and 65%, respectively, and early diagnosis and specific treatment of mothers can reduce the risk of fetal infection up to 50% [9].

In many studies, contaminated drinking water and close contact with cats have been implicated as sources of* Toxoplasma* infection in humans worldwide [10–14]. However no statistically significant association between these risk factors and* Toxoplasma* seropositivity was observed in the current study. In regard to location, the prevalence rate of IgG anti-*Toxoplasma gondii* antibodies in women with abortion was 19/90 (21.1%) and 13/40 (32.5%) for urban and rural cases, respectively, which indicated significant differences (*P* < 0.05). The possible reason for this difference is the more contact with soil in rural individuals in comparison with urban ones.

Diagnosis of* T. gondii* infection before conception is very essential especially in population with low seroprevalence rate but is usually not possible and therefore testing for antibodies to* Toxoplasma* in pregnancy is performed only in suspected cases. This study showed that the prevalence of IgG antibody was 24.6% in women with abortion and 21.5% in the control group; however, this difference was not statistically significant. In addition IgM antibody was detected in one woman who had aborted but not in women with normal childbirth.

Nimri et al. [15] reported that IgG results of the cases differed significantly from those of the controls (54% and 12% resp.; *P* < 0.02). In addition 2.7% of the cases were IgM positive, in the test group.

Ebadi et al. [16] in seroprevalence study in Jahrom, Iran, revealed a higher prevalence of anti-*Toxoplasma* IgG antibody in women with repeated abortions (17.5%) compared with the control group (14%); however, the difference was not statistically significant.

Sharif and Ajami [17] reported the frequency of anti-*Toxoplasma* IgG and IgM in women with a history of abortion or stillbirth in Sari to be 34.21% and 7.89%, respectively. In 2007 [18], in Bandar Abbas, south Iran, 124 women with a history of abortion were evaluated for the frequency of anti-*Toxoplasma* IgG and IgM. It was found that 98 (79.03%) and 19 (15.32%) patients were positive for IgG and IgM, respectively.

Saeedi et al. [19] showed that the frequency of anti-*Toxoplasma* antibodies among the women with normal and abnormal pregnancy was, respectively, 45.5% and 44.1% for IgG (*P* = 0.01) and 46.5% and 21% for IgM (*P* = 0.002), with significant relationship observed only between abortion and IgM titers.

Study conducted in Kashmir by Zargar et al. [20] in 1998 revealed the prevalence of IgM anti-*Toxoplasma* antibody in women with recurrent abortion (49.47%), which was more than the control group with normal delivery (8.88%); they suggested a probable relationship between* Toxoplasma* infection and recurrent abortion. Bobic et al. [21] in 1991 showed that the prevalence of IgG and IgM antibodies in women with uncomplicated abortion is 44.9% and 33.3%, respectively.

## 4. Conclusions

Although no statistical significant differences of IgG anti-*Toxoplasma gondii* prevalence were observed between the case and control groups in this study, detection of IgM antibody in a woman who had abortion indicates the possible role of* Toxoplasma gondii* in abortion phenomenon in the region. Because studies show IgM antibodies may persist for 1 year after infection and the positive result for the antibodies must be interpreted with caution [22, 23]. In order to improve the diagnosis of primary infection with* T. gondii* in pregnancy using anti-*Toxoplasma* IgG avidity and PCR which have the ability to discriminate between recent and prior infections are recommended [24].
